# PLATEAU-PATELLA ANGLE: AN OPTION FOR ASSESSING PATELLAR HEIGHT ON PROXIMAL TIBIA OSTEOTOMY

**DOI:** 10.1590/1413-785220162403158611

**Published:** 2016

**Authors:** MARCELO BATISTA BONADIO, JÚLIO AUGUSTO DO PRADO TORRES, VICENTE MAZZARO, CAMILO PARTEZANI HELITO, RICCARDO GOMES GOBBI, MARCO KAWAMURA DEMANGE

**Affiliations:** 1. Universidade de São Paulo, Faculdade de Medicina, Hospital das Clínicas, Instituto de Ortopedia e Traumatologia, Knee Group, São Paulo, SP, Brazil.

**Keywords:** Patellar ligament, Osteotomy, Knee.

## Abstract

**Objective:**

To compare the plateau-patella angle method to the methods already established for patellar height measurement in patients undergoing high tibial osteotomy.

**Methods:**

This is a retrospective study of 13 patients undergoing medial opening tibial osteotomy. The patellar height was measured in pre and post-operative radiographs by the methods from Insall-Salvati, Caton-Deschamps, Blackburne-Peel and patella-plateau angle, as well as the tibial slope and length of the patellar tendon. Measurements were performed by two knee surgeons at two different times.

**Results:**

The mean age was 41.33 ± 01.09 years old. The average rates of Caton-Deschamps, Blackburne-Peel, Insall-Salvati and plateau-patella angle were, respectively, 1.00; 0.89; 1.10; and 23.15° preoperatively, and 0.89; 0.78; 1.11; and 20.46°, postoperatively. The correlation of Caton-Deschamps, Blackburne-Pell, and Insall-Salvati indexes and plateau-patellar angle interobserver was 0.72 (p <0.001), 0:54 (p <0.001), 0.65 (p <0.001), and 0.67 (w <0.001), respectively.

**Conclusion:**

The plateau-patella angle method undergoes changes that are correlated with changes in tibial slope after osteotomy, unlike the classical methods. This fact may lead to overestimate the reduction of patellar height after osteotomy. ***Level of evidence IV. Case Series.***

## INTRODUCTION

Historically the measurement of patellar height was widely studied for its causal relationship to various diseases of the patella-femoral joint.^1^ ^-^ ^8^ The patella is associated with anterior knee pain, chondromalacia and patellar instability.^1^ ^-^ ^3^ On the other hand, low patella presents association with patellar overpressure and patellofemoral arthrosis.^7^ ^-^ ^9^

After high tibial osteotomies there may be change in the patellar height. One of the first methods to assess patellar height was proposed by Blumensat in 1938,^10^ based on the evaluation of the profile radiograph, with the knee in 30° flexion. Drawing up a radiographic line of the intercondylar ceiling and projecting it anteriorly; it should cross the inferior pole of the patella in height considered normal. If the inferior pole found the top of the line, the patella would be considered high. This method was subsequently considered inaccurate, especially due to the difficulty in obtaining radiographs with perfect 30° flexion, essential for the assessment.^4^ ^,^ ^11^

Radiographic evaluation methods of patellar height most commonly used are those by Caton et al.^12^ Insall-Salvati^13^ and Blackburne-Peel.^4^ These methods involve calculating the ratio between these measurements.

**Figure 1:**
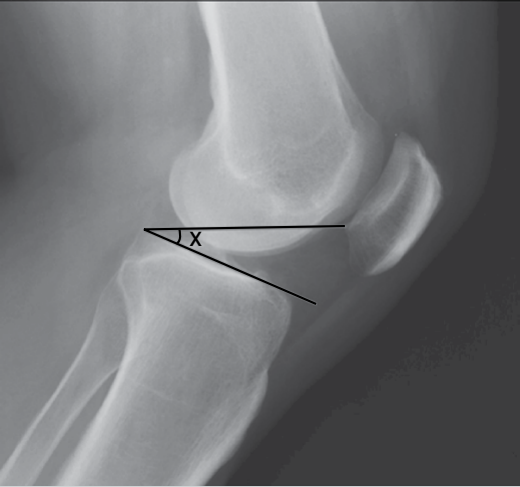
Measurement method of the plateau-patella angle.

In order to eliminate calculations, Portner-Pakzad^14^ proposed a method called "plateau-patellar angle". This method requires only a single angle measurement, eliminating additional calculations. Because it is an angular evaluation, there is no interference due to radiographic magnification and size of the patient, requiring only a minimum 30° knee bending on the radiograph. (Figure 1)

Although there are new validation studies of the method proposed by Portner-Pakzad,^14^ namely plateau-patella angle, it has not been studied as patellar height measurement method in postoperative high tibial osteotomies. The aim of this study is to compare the plateau-patella angle with classic measurement methods of patellar height, and validate it as a measuring method after high tibial osteotomies.

## MATERIALS AND METHODS

We performed a retrospective review of all medial opening osteotomy of the tibia held in a single center from December 2012 to June 2015. Knee profile digital radiographs were assessed immediately before and three months after surgery. Radiographs with inadequate rotation, poor definition of radiographic landmarks, or knee flexion lower than 30° were excluded. From a total of twenty patients, seven were excluded because of inadequate radiographs. This study was approved by the Ethics Research Committee of *Hospital das Clínicas da Universidade de São Paulo* under protocol number 1160.

Thirteen knee radiographs were evaluated from 13 patients who underwent medial opening osteotomy of the proximal tibia. In this sample, one patient was female and 12 were male; there were five right knees and eight left knees. The mean age was 40.8 ± 9.01 years old (maximum, 58 and minimum, 28 years old). Regarding indication for osteotomy, four patients underwent osteotomy for osteoarthritis of the medial compartment of the knee, eight patients for correction of lower limb alignment associated with ligament reconstruction and one patient to decrease the load on the medial compartment due to extensive chondral injury.

The tibial slope was measured on x-rays, the length of the patellar tendon and patellar height were assessed by classical methods from Insall-Salvati, Caton-Deschamps and Blackburne-Peel and also by the plateau-patella angle method. The measurements were performed by two orthopedic specialists in knee surgery, each of them conducted two evaluations at two different times separated by a minimum of 30 days.

All measurements were made according to the original description of their respective autors.^4^ ^,^ ^11^ ^-^ ^13^ The Insall-Salvati index is the ratio between the length of patellar tendon divided by the length of the patella. The tendon length was measured in its deepest portion from its origin at the inside pole of the patella up to its insertion into the anterior tibial tuberosity. The length of the patella was measured in its largest diagonal length.

According to the Blackburne-Peel method,^4^ the perpendicular distance from the inferior end of the articular surface of the patella until the anterior projection of the articular surface line of the tibial plateau was divided by the length of the articular surface of the patella. The Caton-Deschamps index was calculated as the ratio of the distance from the lower end surface of the patella to the tibial supero-anterior angle divided by the length of the articular surface of the patella.

The plateau-patellar angle described in the Introduction was measured between a first line tangent to the medial plateau of the tibia (same line used by Blackburne and Peel^4^ ^)^ and a second line from the posterior end of the medial plateau towards the lower end of the articular surface of the patella.

Ranges were considered normal between 0.6 and 1.2 for the Caton-Deschamps index, 0.8 and 1.2 for Insall-Salvati index and 0.5 to 1 for the Blackburne-Peel^4^ index, and 21° and 29° to the plateau-patella angle.

For qualitative assessment we used a classification as high, normal or low patella, according to previously mentioned normal intervals and compared preoperative to postoperative scores.

### STATISTICAL ANALYSIS

We used the Pearson correlation coefficient to evaluate the association between continuous variables. For the evaluation of differences between pre- and postoperative period in each method we used the paired Student's *t*-test.

For comparison of patellar height changes between pre- and postoperative periods among the various measurements, an assessment of the percentage change was made. We considered as the denominator the difference from the normal range for each method. Therefore, we considered a denominator of 0.6 for Caton-Deschamps index, 0.4 for the Insall-Salvati index, 0.5 for Blackburne-Peel index^4^ and 8° to the plateau-patella angle.

The numerator was the mean change between pre and postoperative. We, therefore, calculated the ratio of the mean change for each method, managing to compare different methods with different reference ranges and allowing the quantitative assessment of the change in patellar height in each method after proximal tibial osteotomy. For comparing the change between the methods we used the paired Student's *t*-test.

## RESULTS

The average rates of Caton-Deschamps, Blackburne-Peel, Insall-Salvati and plateau-patella angle were, respectively, 1.0 / 0.89 / 1.10 and 23.15° preoperatively and 0.89 / 0.78 / 1.11 and 20.46° postoperatively. (Table 1) The mean tibial slope pre and postoperative was, respectively, 7.58° and 9.18° (*p<*0.05). The mean tendon patellar length was, respectively, 502.71mm and 507.46mm (*p* = 0.56), a not statistically significant difference. (Table 1)

**Table 1:** Statistics of pre- and postoperative measurements.

	Mean	Standard deviation	Minimum	Maximum	p value*
Caton-Deschamps					
Preop	1.00	0.17	0.69	1.37	
Postop	0.89	0.18	0.48	1.22	0.0000019
Blackburne-Peel					
Preop	0.89	0.17	0.56	1.27	
Postop	0.78	0.17	0.47	1.12	0.0000009
Insall-Salvati					
Preop	1.10	0.15	0.83	1.50	
Postop	1.11	0.12	0.81	1.41	0.6892418
Plateau-patella angle					
Preop	23.15	3.03	15.00	31.12	
Postop	20.46	2.72	14.00	25.58	0.0000007
Tibial slope					
Preop	7.58	4.38	-1.00	16.80	
Postop	9.18	4.54	-1.00	19.50	0.0000226
Patellar tendo length					
Preop	502.71	63.33	373.00	656.00	
Postop	507.46	60.19	324.00	662.00	0.5689496

*p value: Student's t-test for difference between pre and postoperative measurements

The absolute mean change and percentage of each method were -0.11 (18%); 0.01 (2%), -0.11 (-22%) and -3.01° (-38%), respectively, for Caton-Deschamps, Blackburne-Pell, Insall-Salvati and plateau-patella angle. (Table 2) The difference in the percentage change at pre and postoperative was significant between the Insall-Salvati index and other methods and between the plateau-patella angle and the other methods. (Table 3)

**Table 2:** Differences between pre and postoperative measurements.

Differences between pre and postoperative	Mean	%	Standard Deviation
Caton-Deschamps	-0.11	-18%	0.15
Insall-Salvati	0.01	2%	0.13
Blackburne-Peel	-0.11	-22%	0.14
Plateau-patella angle	-3.01	-34%	3.43

**Table 3:** Correlation between methods to assess patellar height.

Comparison	p value*
Caton-Deschamps x Insall-Salvati	0.0000746
Caton-Deschamps x Blackburne-Peel	0.2607340
Caton-Deschamps x Plateau-patella angle	0.0176416
Insall-Salvati x Blackborne-Peel	0.0000735
Insall-Salvati x Plateau-patella angle	0.0000127
Blackburne-Peel x Plateau-patella angle	0.0158460

*p value: paired t-test

The correlation between the change of the plateau patella angle between the pre- and postoperative period and the change of the slope after surgery was -0.67 (*p<*0.001).

Regarding qualifying assessment of methods, we noticed a change trend to a lower patellar height postoperatively compared to preoperative. For Caton-Deschamps index, 7.7% of preoperative ratings changed to a lower height in the postoperative period, for Insall-Salvati index also 7.7%, for Blackburne-Peel index 19.2%, and plateau-patella angle 46.2%.

The correlation of Caton-Deschamps, Blackburne-Pell, Insall-Salvati indexes and intra-observer plateau-patellar angle were, respectively, 0.91 (*p<*0.001), 0.86 (*p<*0.001), 0.84 (*p<*0.001), 0.81 (*p<*0.001) for observer one and 0.93 (*p<*0.001), 0.96 (*p<*0.001), 0.92 (*p<*0.001) and 0.98 (*p<* 0.001) for observer two. The inter-observer correlation coefficient was 0.72 (*p<*0.001), 0.54 (*p<*0.001), 0.65 (*p<*0.001) and 0.67 (*p<*0.001), respectively.

## DISCUSSION

Phillips et al.^14^ concluded, in his review in 2010, that there is clear room for improvement in existing evaluation methods of patellar height available at that time. The three most classical methods of measuring patellar height presented some difficulties. The Insall-Salvati index can be changed by a patella with a larger non-articular portion,^3^ and it is often difficult to precise the exact location of insertion of the patellar tendon. The method, thus, depends on a perfect knee profile. On the other hand, the Blackburne-Peel^4^ index does not use the length of the patellar tendon, but also depends on mathematical calculation, building lines and more complex measurements. Regarding Caton-Deschamps index, identifying the superior-anterior tibial angle presents a grid variability in patients with osteoarthritis and this figure becomes even more difficult to assess.^9^ The plateau-patella angle is a simpler method, which avoids mathematics calculations for patellar height measurement, with easier to identify radiographic landmarks, already validated by Portner et al.,^14^ but also depends on absolute profile x-rays with at least 30° flexion. In the present study the method showed good intra- and interobserver correlation, demonstrating that the simplicity of the angle improves its reproducibility.

The proximal osteotomy of the tibia tends to change the patellar height and the medial opening osteotomy, specifically, has a tendency to lower the patella, a finding confirmed in this study.^15^ However, the extent of height variation can change between the different methods of patellar height assessment. The plateau-patella angle is an already validated method to measure patellar height,^13^ however, to our knowledge, there is study comparing traditional measuring methods of patellar height in patients undergoing medial opening osteotomy of the tibia.

Our results showed a clear trend of reduction of patellar height after medial opening osteotomy of the tibia, except only regarding Insall-Salvati index, which showed no significant change in patellar height after surgery. This exception can be explained by the fact that Insall-Salvati uses the length of the patellar tendon and patellar diameter to calculate its index and none of those parameters is directly changed with the surgery. Thus, the Insall-Salvati index is not prone to elevation of the articular line that occurs with the medial opening osteotomy and does not identify the reduction in the patellar height in these cases. Therefore, we believe that the method should not be used in the evaluation of patients submitted to this surgery.

The reduction of patellar tendon length resulting from fibrosis, despite having already been demonstrated in previous studies,^16^ was not found in our evaluation. Since we used x-rays performed only 3 months after surgery, it is still possible that some variation may have occurred regarding the shortening of the tendon.

Assessing the three methods that demonstrated changes of the patellar height after surgery, Blackburne-Peel index,^4^ Caton-Deschamps index and plateau-patella angle, the change is more significant on the plateau-patella angle. Considering the normal range of each of the methods, the percentage change was 22%, 18% and 34%, respectively. But only the plateau-patella angle method showed significance when compared to the others, showing that the method has a greater change after tibial high osteotomy as compared to conventional methods. This difference was also observed in the qualitative evaluation, in which we observed a change of 46.2% on the preoperative ratings as compared to postoperative by the plateau-patella angle.

Another change observed after medial opening osteotomy in the study was the increase of tibial slope. Theoretically, the tibial slope should not be changed with this type of osteotomy, unless the surgeon seeks to protect the Anterior Cruciate Ligament or Posterior Cruciate Ligament with slight slope variations. But this finding is not uncommon, being one of the most prevalent technical errors, and the surgeon should always pay attention to gross changes that may generate significant mechanical changes in the knee.

When we associate these two postoperative changes, alteration of the plateau-patella angle and the change of the tibial slope, we found a significant correlation of -0.67. Thus, we found that the slope increase after the osteotomy has a significant correlation with the reduction of the plateau-patellar angle. Therefore, although the plateau-patella method follows the same trend of reduction of patellar height methods of Caton-Deschamps and Blackburne-Pell, perhaps this one overestimates this reduction, due to changes in tibial slope after osteotomy.

In evaluating the correlation coefficients, all methods achieved a quite satisfactory intra-observer correlation. Regarding the inter-observer correlation, the Blackburne-Peel^4^ method had the worst coefficient (0.54), while the plateau-patella angle obtained a 0.67 index, quite similar to that achieved by Caton-Deschamps method that best correlated between observers, with a coefficient of 0.72. These values demonstrate an adequate reproducibility of the method, probably due to its simple implementation.

The small number of patients included in the study is an important limitation of this study, mainly caused by the need of perfect radiographs for the application of the four methods studied. Future prospective studies with more rigorous x-rays, with a larger number of patients, may bring new information.

## CONCLUSION

We conclude that for patients undergoing high tibial osteotomy, the plateau-patella angle method undergoes changes that alter the tibial slope, with major changes after osteotomy when compared to conventional methods used to assess patellar height. Therefore, its use in patients undergoing this type of procedure can overestimate the reduction of patellar height.
